# Video-Delivered Family Therapy for Perinatal Women With Depressive Symptoms and Family Conflict: Feasibility, Acceptability, Safety, and Tolerability Results From a Pilot Randomized Trial

**DOI:** 10.2196/51824

**Published:** 2023-11-03

**Authors:** Fallon Cluxton-Keller, Mark T Hegel, Craig L Donnelly, Martha L Bruce

**Affiliations:** 1 Department of Psychiatry Geisel School of Medicine at Dartmouth Lebanon, NH United States

**Keywords:** perinatal depression, family conflict, family therapy, family, conflict, depression, depressive, perinatal, pregnant, pregnancy, video, videos, feasibility, safety, acceptability, tolerability, tolerable, families, satisfaction, resilience, psychotherapy

## Abstract

**Background:**

Although individual-level treatments exist for pregnant and postpartum women with depression, family conflict is a significant factor that can contribute to the development and severity of perinatal depressive symptoms. Yet, there is a lack of research on family therapy for perinatal women with moderate to severe depressive symptoms and family conflict. Further, research is needed on the feasibility, acceptability, safety, and tolerability of family therapies for perinatal depression that are delivered using Health Insurance Portability and Accountability Act–compliant videoconferencing technology (VCT).

**Objective:**

This paper describes the feasibility, acceptability, safety, and tolerability of a VCT-based family therapeutic intervention, Resilience Enhancement Skills Training (REST), for perinatal women with moderate to severe depressive symptoms and moderate to high conflict with their family members.

**Methods:**

This paper includes data from an ongoing randomized trial that compares an experimental family therapeutic intervention (REST) to standard of care (VCT-based problem-solving individual therapy) for the treatment of moderate to severe depressive symptoms in perinatal women with moderate to high family conflict. Both interventions were delivered by masters-level therapists using VCT. A total of 83 perinatal women and their adult family members (N=166 individuals) were recruited for participation in the study. Feasibility, defined as therapist adherence to ≥80% of REST session content, was assessed in audio-recorded sessions by 2 expert raters. Acceptability was defined as ≥80% of families completing REST, including completion of ≥80% homework assignments and family report of satisfaction with REST. Completion of REST was assessed by review of therapist session notes, and satisfaction was assessed by participant completion of a web-based questionnaire. The Beck Depression Inventory-Second Edition was administered to perinatal women by research assistants (blind to study group assignment) to assess safety, defined as a reduction in depressive symptoms during the treatment phase. The Family Environment Scale-Family Conflict subscale was administered by therapists to participants during the treatment phase to assess tolerability, defined as a reduction in family conflict during the treatment phase.

**Results:**

On average, the therapists achieved 90% adherence to REST session content. Of the families who started REST, 84% (32/38) of them completed REST, and on average, they completed 89% (8/9) of the homework assignments. Families reported satisfaction with REST. The results showed that REST is safe for perinatal women with moderate to severe depressive symptoms, and none discontinued due to worsened depressive symptoms. The results showed that REST is well tolerated by families, and no families discontinued due to sustained family conflict.

**Conclusions:**

The results show that REST is feasible, acceptable, safe, and tolerable for families. These findings will guide our interpretation of REST’s preliminary effectiveness upon completion of outcome data collection.

**Trial Registration:**

ClinicalTrials.gov NCT04741776; https://clinicaltrials.gov/ct2/show/NCT04741776

## Introduction

### Background

The Maternal, Infant, and Early Childhood Home Visiting Program funds States, territories, and tribal entities to develop and implement evidence-based home visiting in an effort to improve many outcomes including maternal mental health and family function [1]. Many research studies have shown that over a third of home visited mothers (pregnant and post-delivery) screen positive for depression [2-4]. Although home visitors refer depressed mothers for treatment, they infrequently complete it due to barriers (eg, no childcare and no transportation) [5,6] that are compounded for those living in rural areas of the United States [7,8]. Moreover, research has shown that family conflict contributes to depressive symptom severity in home visited mothers [9-15]. Although there is a need to address conflict in home visited families with mothers with moderate to severe depressive symptoms (eg, [9-14]), evidence-based family therapy treatments do not yet exist for them. For this reason, Resilience Enhancement Skills Training (REST) was first pilot-tested in 2017 as a Health Insurance Portability and Accountability Act–compliant videoconferencing technology (VCT)–based family therapeutic treatment to bypass barriers to treatment access for home visited mothers in rural areas with clinically significant depressive symptoms and family conflict [16,17]. REST is based on general systems theory [18] and dialectical behavior therapy (DBT) skills training [19]. The pandemic magnified a critical need for accessible and effective VCT-based treatments for home visited mothers [20]. Yet, the research is limited on the feasibility, acceptability, safety, and tolerability of VCT-based treatments for perinatal individuals with depression, including home-visited mothers.

### Prior Work

REST’s acceptability, safety, and tolerability were explored in 2 implementation-effectiveness hybrid pilot trials [16,21]. The first study included 13 home visited families (N=26 individuals) and a historical comparison group of 13 home visited depressed mothers who refused treatment [16]. The second study included 24 couples (N=48 individuals) recruited from rural obstetrics clinics but did not include a comparison group [21]. Although a few mothers with mild depressive symptoms enrolled in the previous trials, the majority of the mothers had moderate to severe depressive symptoms at baseline [16,21]. Since REST includes DBT skills effective for people with severe depressive symptoms [22-24], it was not surprising that the previous 2 trials of REST showed that mothers with moderate and severe depressive symptoms had the greatest reduction in depressive symptoms [16,21]. Thus, this study only enrolled mothers with moderate to severe depressive symptoms.

### Purpose of the Paper

The research presented in this paper builds on our prior research [16,21,25] to confirm REST’s feasibility, acceptability, safety, and tolerability in home visited families. To date, the current effectiveness-implementation hybrid type 1 trial is the most rigorous study of REST since it is a randomized trial with an active comparison condition and outcome assessors who are blinded to study group assignment. Since this paper focuses on the properties of REST, we primarily describe data from that arm of the trial. We hypothesized that (1) feasibility will be demonstrated by therapist adherence to ≥80% REST session content; (2) acceptability will be demonstrated by ≥80% families completing REST, including completion of ≥80% of homework assignments and family report of satisfaction with REST; (3) safety will be demonstrated by reductions in maternal depressive symptoms during the treatment phase; and (4) tolerability [26,27] will be demonstrated by reductions in family conflict during the treatment phase.

## Methods

### Ethical Considerations

This effectiveness-implementation hybrid trial has been approved by an institutional review board (IRB; 2000691) at an academic medical center in New England. Study participants provided consent for the eligibility screen and study enrollment, including consent for audio-recorded sessions.

### Recruitment

The recruitment strategy for this trial was established with the aim to recruit a representative sample and achieve participant enrollment benchmarks. Study participants were recruited into the randomized trial from participating home visiting agencies. The study was conceptualized prior to the COVID-19 pandemic and implemented during the pandemic. Thus, some of our original study implementation strategies (in-person home visiting staff training and in-person consent) required a transition to web-based formats due to the pandemic. Home visitors used standard depression screening and referral procedures to refer mothers to the study for the eligibility screen [16]. Home visitors read an IRB-approved study information sheet to mothers who screened positive for depression and obtained consent from those who requested to be referred to research staff to learn more about the study. The recruitment strategy was an iterative process since the originally proposed plan had to be revised due to the COVID-19 pandemic and further tailored to broadly address differences in agency staff culture and family culture. For instance, many of the participating home visiting agencies transitioned to VCT-based home visits, using VCT, during the pandemic, but some home visitors presented the IRB-approved study information sheet to some families in person due to the family’s sensitivity to topics related to mental health.

A total of 236 individuals were screened for eligibility from April 2021 through March 2023. Of the 236 individuals, 46 of them did not meet the eligibility criteria (described in the next subsection) and 24 eligible individuals decided not to enroll in the study primarily due to busy schedules or upcoming moves to different locations. Thus, a total of 83 families (N=166 individuals) enrolled in the study (REST: n=84 individuals and VCT-based problem-solving individual therapy [V-PST]: n=82 individuals).

### Eligibility Criteria

Each family is a dyad that includes the mother and her adult relative or her current intimate partner. Mothers met the following inclusion criteria: (1) enrolled in a participating home visiting agency; (2) in any trimester of pregnancy and up to 18 months postpartum; (3) at least 15 years; (4) fluent in English with at least an eighth-grade education since intervention materials are written in English for this level of education; (5) Beck Depression Inventory-Second Edition (BDI-II) [28] scores of at least 20, indicative of moderate to severe depressive symptoms; (6) Perceived Hostility Survey (PHS) ages 18+ years [29] raw scores of at least 16 [30] or PHS ages 15-17 years old raw scores of at least 14 [30], indicative of moderate to high conflict with the selected family member with whom they live in the same home or have at least weekly contact; and (7) have consistent internet access on a cell phone, tablet, or computer with a working camera and microphone [25]. Mothers with current domestic violence in their homes or histories of domestic violence with the selected family member on the Abuse Assessment Screen were ineligible for participation in this study [25]. The study includes a detailed protocol for mothers who report domestic violence, and they are provided with emergency assistance by local service providers who address domestic violence [25]. Mothers’ family members met the following inclusion criteria: (1) mother’s adult relative or her current intimate partner, (2) fluent in English with at least an eighth-grade education, (3) PHS ages 18+ years raw scores of at least 16 or PHS ages 15-17 years old raw scores of at least 14 with the mother, and (4) consistent internet access on a cell phone, tablet, or computer with a working camera and microphone [25].

### Overview of Study Interventions

#### Resilience Enhancement Skills Training

REST includes a total of 10 weekly, 45-minute, family therapy sessions delivered by a mental health professional using VCT. In this study, 2 masters-level therapists delivered REST to participants. REST is informed by family-oriented DBT skills training [19] and general systems theory [18]. REST is not a parenting skills program [25]. Similar to the DBT skills training model [19], REST uses a psychoeducational format. REST includes the following DBT core skills: mindfulness, distress tolerance, emotion regulation, and interpersonal effectiveness [19]. REST has been described in detail in other papers [16,21,25]. REST’s flexible skill application allows therapists to help families, regardless of the configuration, to apply skills to aspects of their relationships based on each family’s preferences. Therapists ask families specific questions about how they want to apply the skills to their relationship and assist each family in developing a plan to practice the skills in the areas to which the family deems the skills are applicable. For example, a mother and her grandmother use interpersonal effectiveness skills to set boundaries related to time management, or a mother and her intimate partner use interpersonal effectiveness skills to negotiate differences pertaining to finances.

#### VCT-Based Problem-Solving Individual Therapy

V-PST includes a total of 10 weekly, 45-minute, individual therapy sessions for only the mother that are delivered by a mental health provider using VCT. In this study, masters-level therapists delivered V-PST. V-PST is a structured approach that is based on cognitive behavioral therapy [31]. PST is considered a standard of care since it is an evidence-based treatment for moderate and severe depression in adolescents (age 15 years and older) and adults [32,33].

### Overview of REST Training for Therapists

The first author trained REST therapists to implement it with families in a VCT-based session that included 2 parts. Part 1: 2 hours of didactic instruction on the REST Therapist Manual and practice exercises for each REST module and part 2: 4 hours of role-plays with feedback to allow therapists to demonstrate the use of the REST Therapist Manual, knowledge of REST skills, and skill application in different family scenarios [25]. The first author provided feedback to therapists during role-plays and supervised REST therapists on a weekly basis [25].

### VCT Training for Therapists and Study Participants

Therapists used VCT, WebEx (healthcare version; Cisco Systems Inc), to deliver V-PST and REST in this study. The technology requirements for therapists include a computer with a working microphone and camera, a word processing program to open skills documents for participants to view during sessions, and consistent, secure internet access (eg, ethernet connection) with a WebEx account [25]. Technology requirements for participants include consistent internet access (eg, subscription to an internet service provider) on an electronic device (eg, cell phone, tablet, or computer) with a working camera and microphone along with the WebEx app to participate in sessions [25]. The VCT training details for therapists and study participants were published in a previous paper [25] and are summarized in this subsection. Each therapist underwent training on how to download and install VCT software and use the camera, microphone, screen share, and audio record features [25]. The average length of the VCT training was about 23 minutes. Therapist knowledge of VCT was assessed by the first author through observation of their use of the audio, video, screen share, and recording features on their computers during the training [25]. In addition, a troubleshooting technology guide was provided to each therapist to assist participants in navigating camera and audio interruptions due to incoming phone calls and low bandwidth [25]. Each study participant received individualized training on how to download and install the VCT software and use of the camera and audio features [25]. The average length of VCT training for study participants was 10 minutes. Study participant knowledge of VCT was assessed by the first author through observation of use of the audio and video features on their electronic devices (cell phone, tablet, or computer) [25].

### Feasibility

Feasibility is defined as therapist adherence to ≥80% of REST session content [25]. Fidelity monitoring details were published in a previous paper [25] and are summarized in this subsection. The first author developed the REST fidelity measure to rate therapist adherence to REST session content in the audio-recorded sessions, and the authors (FC-K and MTH) used the measure to monitor fidelity. These 2 authors rated therapists on the quality of REST content delivery in 73 audio-recorded sessions using a scale that ranged from 0 to 2 (0: no content delivered, 1: partial content delivered, and 2: all content delivered) [25]. Cohen κ was used to assess interrater reliability of the REST fidelity measure, and the authors established sufficient interrater reliability (κ=0.91) [25].

The study included a 2-phase fidelity monitoring process for REST [25]. In phase 1, authors (FC-K and MTH) assessed fidelity in audio-recorded sessions for the first 2 families assigned to each REST therapist, and any discrepancies were discussed and resolved in these fidelity assessments [25]. In phase 2, an author (MTH) assessed fidelity in 10% of randomly selected audio-recorded REST sessions that were originally assessed by the first author [25]. The level of fidelity was continuously assessed to ensure that each therapist achieved at least 80% on the REST fidelity measure for each session, and data from these ongoing assessments were used in weekly supervision [25].

### Acceptability

Acceptability is defined by ≥80% of families attending all 10 REST sessions, including completion of ≥80% of homework assignments and family report of satisfaction with REST. Data were collected by the first author on participant session attendance and homework completion from the therapists’ session notes [25]. The session notes did not contain identifying information about the participants, and all notes were stored on a password-protected secure site within the medical center network [25]. Participants were emailed links to complete a web-based satisfaction questionnaire [16] in Research Electronic Data Capture (REDCap; Vanderbilt University) within a week after the final REST session. The satisfaction questionnaire includes items on the usefulness of the REST skills and VCT as a delivery method and semistructured open-ended free-text items on the helpfulness of REST skills and changes to REST [16]. The satisfaction questionnaire demonstrated strong reliability (α=.85) in our previous study [16] and this study (α=.82).

### Safety

Safety was defined as a reduction in maternal depressive symptoms on the BDI-II during the treatment phase, and ≤5% of mothers discontinuing REST due to worsening depressive symptoms. The BDI-II [28] was used to monitor the safety of mothers in both study groups. Research assistants, blinded to study group assignment, verbally administered the BDI-II, via VCT or by phone (depending on the mother’s preference), to mothers at sessions 2, 4, 6, and 8 time points during the treatment phase. These time points align with standard guidelines for assessing moderate to severe perinatal depressive symptoms [34,35]. Consistent with treatment research on depressive symptom deterioration [36,37] and safety monitoring protocols for perinatal depression intervention research [34,36-39], the protocol required expedited referrals for local mental health services for mothers with an increase of 9 points on the BDI-II in the treatment phase. We established a requirement for expedited referral to the study psychiatrist [39,40] by converting an Edinburgh Postnatal Depression Scale score of 21, used in primary care settings, to a BDI-II score of 40 [40,41]. The safety protocol required expedited referral to the study psychiatrist for mothers who maintain BDI-II scores of ≥40, with no point reduction, for two consecutive time points in the treatment phase and mothers who report suicidal thoughts in the treatment phase. This study also includes a detailed protocol for mothers who report suicidal ideation on the BDI-II item 9 (rating of 2 or 3), and they were provided with emergency assistance [25].

### Tolerability

Tolerability [26,27] is defined as a reduction in family conflict in the treatment phase and low discontinuation rates (≤5% of families) of REST due to sustained family conflict. Therapists verbally administered the Family Environment Scale-Conflict subscale (FES-C) [42] to each mother and her family member, separately, at the end of sessions 4 and 8 using VCT or by phone (depending on the participant’s preference). Sustained family conflict was defined as no change or an increase in FES-C scores, from baseline, at either time point in the treatment phase (session 4 or session 8).

Per the tolerability protocol, the first author would contact families who discontinued REST to determine if they discontinued it due to sustained family conflict and provide them with appropriate referrals to providers in the community. The FES-C includes an item on domestic violence, and the study includes a detailed protocol for participants who report domestic violence as they are provided with emergency assistance by local service providers who address domestic violence [25].

### Analytic Plan

Univariate statistics were used to characterize REST therapists and participants at baseline by study group.

#### Feasibility

For therapist adherence to REST, the mean adherence score (percent) for delivery of session content was calculated using the average adherence score divided by the total possible score [25]. The number of sessions that included a lack of adherence to REST and deviations were calculated, and the reasons were recorded [25].

#### Acceptability

Quantitative and qualitative analyses were conducted to assess acceptability.

##### Quantitative Analysis

The average number of attended therapy sessions was calculated for mothers and family members separately, and the percent of attended sessions was calculated out of 10. Data on completed homework assignments were collected by the first author through a review of therapist session notes and content from audio-recorded sessions. The percent of completed homework assignments was calculated overall. Univariate statistics were used to summarize responses to the satisfaction questionnaire multiple-choice items.

##### Qualitative Analysis

A thematic analytic approach was used to code the semistructured open-ended questions on the satisfaction questionnaire [16,43]. Codes were grouped into categories to create themes, and the emergent themes were identified and summarized [43]. For the question on the most helpful skill, numeric values were assigned to each theme, and themes were rank-ordered by the number of written responses for each type of skill [16].

#### Safety

Changes in BDI-II scores were calculated at each time point to closely monitor the safety of each mother in each study group. We used V-PST mothers’ BDI-II scores as reference scores to determine if patterns of change in REST mothers’ BDI-II scores were similar. Univariate statistics were used to summarize changes in maternal BDI-II scores during the treatment phase (session 2, 4, 6, and 8 time points) for mothers in each study group.

#### Tolerability

The FES-C score for each mother and her family member was calculated separately for the session 4 and session 8 time points. Descriptive statistics were used to analyze tolerability by participant type (mother or family member) for those randomized to REST.

## Results

### REST Therapist Characteristics

Two female master-level therapists delivered REST. One therapist identified as more than 1 race, and the other therapist identified as Caucasian [25]. Each had over 10 years of experience working with home visited families [25]. Each therapist had knowledge of general systems theory and cognitive behavioral family therapies [25].

### Participant Characteristics

The baseline psychosocial characteristics of the study population are included in Table 1. In total, 60 out of 83 mothers (72%) selected their current intimate partners to participate with them in the study. There were no significant differences in family member type (eg, mother’s adult relative and mother’s intimate partner) by maternal age. Overall, 7 out of 21 (33%) pregnant mothers were pregnant with their first child, and 25 out of 83 mothers (30%) were married at baseline. On average, mothers in both study groups had moderately severe depressive symptoms on the BDI-II at baseline (REST BDI-II: mean 28.4, SD 5.9 and V-PST BDI-II: mean 27.0, SD 6.2).

**Table 1:** Baseline family characteristics by study group (N=166).

		REST^a^		V-PST^b^	
		Mothers (n=42)	Family members (n=42)	Mothers (n=41)	Family members (n=41)
Age (years), mean (SD)		28.1 (5.7)	33.1 (11.4)	28.3 (5.5)	38.0 (14.6)
**Race, n (%)**					
	American Indian	1 (2)	1 (2)	3 (7)	2 (5)
	White	28 (67)	25 (59)	28 (68)	25 (61)
	More than 1 race	9 (21)	12 (29)	7 (17)	11 (27)
	Other races	4 (10)	4 (10)	3 (7)	3 (7)
Pregnant, n (%)		11 (26)	1 (2)	10 (24)	0
Family conflict, mean^c^		58.7	60.1	60.1	60.1
**Highest level of education, n (%)**					
	8th through 12th grade	8 (19)	6 (14)	4 (10)	5 (12)
	High school graduate	15 (36)	19 (46)	19 (46)	17 (42)
	Vocational school degree	2 (5)	0	4 (10)	3 (7)
	Some college	9 (21)	9 (21)	9 (22)	7 (17)
	College degree	8 (19)	8 (19)	5 (12)	9 (22)
Employed or student, n (%)		12 (29)	34 (81)	15 (37)	34 (83)

^a^REST: Resilience Enhancement Skills Training.

^b^V-PST: VCT-based problem-solving individual therapy.

^c^NIH Toolbox Perceived Hostility Survey uncorrected *T*-score [29].

### Attrition

Of the 166 participants who enrolled in the study, 16 of them dropped out of the study before starting the assigned intervention (REST or V-PST). Of the remaining 150 participants, 7 REST participants and 11 V-PST participants discontinued the study. Thus, the final sample size is 132 participants (REST: n=70 and V-PST: n=62).

### Feasibility

On average, the therapists achieved 90% adherence to REST session content. One therapist experienced some technological problems with her computer in sessions with the first family to which she was assigned, which resulted in an adherence score below 80% in the first 4 sessions with the family [25]. The therapist delivered content that was missed in the first 4 sessions to the family in subsequent sessions [25]. After the therapist’s computer was fixed, she achieved 90% adherence to REST in sessions with the second and third families to which she was assigned [25]. As previously reported, deviations from some of the REST session content occurred in 7 sessions (1 session per family), which resulted in session adherence scores below 80% [25]. Lack of adherence to some of the content occurred when participants arrived over 15 minutes late and older children interrupted sessions with requests for privileges (ie, requests to play video games) [25]. Therapist adherence to REST did not vary by family configuration.

### Acceptability

As previously mentioned, 7 participants discontinued REST. They discontinued REST due to moving out of the geographical area or employment or increased work hours. On average, mothers attended 9.6 sessions and their family members attended 8 sessions. Of the 38 families who started REST, 32 families (84%) completed REST and 1 family reported significant improvements after only 4 sessions. On average, families completed 89% (8/9) of the homework assignments.

Although each participant was emailed a unique link to independently complete the satisfaction questionnaire, we learned that 12 family members jointly answered the questions using only the mother’s link to the questionnaire. For this reason, the completion rates differed for mothers (n=28) and family members (n=12). Of the 32 families who completed REST, 4 families and 4 family members did not complete the satisfaction questionnaire after multiple email reminders were sent. Overall, families who completed the satisfaction questionnaire reported they were satisfied with REST. In total, 6 participants reported that they would prefer in-person delivered REST sessions, instead of VCT-based REST sessions, in the future. Of these 6 participants, 2 of them reported having some difficulties with accessing the VCT and using the audio feature for some sessions.

For the satisfaction questionnaire free-text item on the most helpful REST skills, 22 out of 40 (55%) written responses included 2 or more types of REST skills that were listed as most helpful. Mindfulness was listed as one of the most helpful types of skills in 45% (18/40) of written responses. Emotion regulation was listed as one of the most helpful types of skills in 30% (12/40) of written responses, and interpersonal effectiveness was listed as one of the most helpful types of skills in 30% (12/40) of written responses followed by distress tolerance in 28% (11/40) of written responses. For the satisfaction questionnaire item on changes to REST, 3 participants reported that they would have liked more sessions, and 1 participant preferred fewer mindfulness exercises.

### Safety

**Figure 1:**
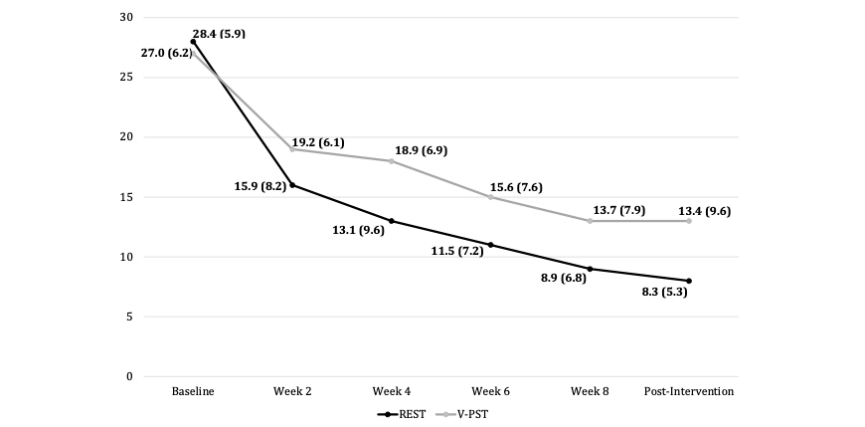
Safety: changes in maternal Beck Depression Inventory-Second Edition scores by time point and study group. REST: Resilience Enhancement Skills Training; V-PST: VCT-based problem-solving individual therapy.

Figure 1 includes the maternal BDI-II scores at baseline, during the treatment phase (session 2, 4, 6, and 8 time points), and at post-intervention (within 1 week after the last session) by study group. Since V-PST is considered a standard of care, we used the V-PST mothers’ BDI-II scores as reference scores to determine if similar patterns of change in depressive symptoms occurred in REST mothers. As shown in Figure 1, mothers in both study groups reported significant reductions in depressive symptoms with greater reductions reported by REST mothers during the treatment phase and at post-intervention. Seven mothers missed 1 clinical monitoring time point due to giving birth, physical illness (eg, COVID-19), or work. Four mothers missed 2 clinical monitoring time points due to work or relocations. One mother missed 3 clinical monitoring time points due to COVID-19 and work.

### Tolerability

REST mothers’ FES-C scores decreased by 1.9 points from baseline (mean 3.1, SD 1.3) during the treatment phase (session 8: mean 1.2, SD 1.4). REST mothers’ family members’ FES-C scores decreased by 1.7 points from baseline (mean 2.7, SD 1.1) during the treatment phase (session 8: mean 1, SD 1.3). Thus, no families discontinued REST due to sustained family conflict. In addition, REST mothers reported an 8-point reduction on the PHS ages 18+ years from baseline to post-intervention (PHS uncorrected *T*-score 46.3), and their family members reported a 7-point reduction on the PHS ages 18+ years from baseline to post-intervention (PHS uncorrected *T*-score 49.8).

## Discussion

### Principal Findings

Our results support our 4 hypotheses. Feasibility was demonstrated by therapist adherence to ≥80% REST session content; on average, therapists achieved 90% adherence to REST session content. This finding suggests that REST can be delivered with high fidelity by community therapists. Although promising, fidelity to REST will need to be monitored by newly trained therapists. Acceptability was demonstrated since 84% (32/38) families completed REST, including completion of 89% (8/9) of homework assignments, and participants reported satisfaction with REST. Reasons for incomplete homework assignments were largely centered on physical illness (eg, COVID-19) in participants or their children. Safety was demonstrated by reductions in maternal depressive symptoms during the treatment phase, and no mothers discontinued REST due to worsening depressive symptoms. Thus, the results show that REST is safe for mothers with moderate to severe depressive symptoms. Tolerability was demonstrated by reductions in family conflict during the treatment phase, and no families discontinued REST due to sustained family conflict. Thus, REST is well tolerated among home visited families.

### Comparison to Prior Work

This study differed from our 2 previous studies of REST, in which the first author delivered REST since it assessed the feasibility of delivery of REST by masters-level community therapists. REST’s acceptability, safety, and tolerability were explored in 2 previous trials [16,21], and these findings can be compared to our findings from this study. In our first study of REST, mothers attended all sessions, and their family members attended an average of 8.4 sessions [16]. In our second study, mothers attended an average of 9.8 sessions, and their family members attended an average of 9.4 sessions [21]. In this study, mothers attended an average of 9.6 sessions, and their family members attended an average of 8 sessions. Thus, maternal and family member session attendance was similar across all 3 studies. Overall, families have reported satisfaction with REST in our studies [16,21]. Participants in the previous studies did not suggest any changes to REST [16]. In this study, 3 participants reported a preference for more sessions, and 1 participant reported a preference for fewer mindfulness exercises. No mothers in any of our studies have discontinued REST due to worsening depressive symptoms [16,21]. Further, no families in any of our studies have discontinued REST due to sustained family conflict [16,21].

### Limitations

This study included 2 notable limitations. First, it was not possible to assess sustained fidelity due to the duration of the intervention phase of the study. We aim to conduct a larger study of REST that will be longer in duration to assess fidelity among a greater number of therapists and to identify factors that contribute to the sustained fidelity of REST. Second, 10 families jointly responded to the satisfaction questionnaire items using only the mother’s web-based link for it. Although each participant was instructed to independently complete this questionnaire and emailed a separate link to complete it, these families did not independently respond to the items, and it is unclear if the answers would be different under these conditions.

### Conclusions

Evidence of REST’s feasibility, acceptability, safety, and tolerability serves as an important guide for the translation of research to clinical practice. We have identified feasible and acceptable strategies to facilitate REST fidelity among community therapists and engage home visited families. Thus, our findings provide essential knowledge on REST’s potential scalability in order to test its effectiveness in a larger-scale clinical trial. Further, these findings will be used to guide the interpretation of REST’s preliminary effectiveness to inform a larger trial. Outcome data collection on REST’s preliminary effectiveness is still underway, and we plan to publish these findings in a subsequent paper.

## Appendix

Multimedia Appendix 1 CONSORT-eHEALTH checklist (V 1.6.1).

## Abbreviations

BDI-II: Beck Depression Inventory-Second Edition
DBT: dialectical behavior therapy
FES-C: Family Environment Scale-Conflict
IRB: institutional review board
PHS: Perceived Hostility Survey
REDCap: Research Electronic Data Capture
REST: Resilience Enhancement Skills Training
VCT: videoconferencing technology
V-PST: VCT-based problem-solving individual therapy
